# Supplementary material for: Rapid turnover of life-cycle-related genes in the brown algae
Source: Genome Biol. 2019 Feb 14;20:35. doi: 10.1186/s13059-019-1630-6 (PMC6374913; doi:10.1186/s13059-019-1630-6)
Supplement: Supplementary file 3 — Protein alignments, corresponding Gblocks html file and CDS sequences for the lineage conserved gametophyte-biased genes. (ZIP 2109 kb) [file 13059_2019_1630_MOESM3_ESM.zip › Con_GA_alignments/Ec-02_006140.aa_ali.fasta-gb.htm]

Ec-02\_006140.aa.fasta.trans.aa\_ali.fasta


## Gblocks 0.91b Results

Processed file: **Ec-02\_006140.aa.fasta.trans.aa\_ali.fasta**  
Number of sequences: **2**  
Alignment assumed to be: **Protein**  
New number of positions: **413** (selected positions are underlined in blue)

```
                         10        20        30        40        50        60
                 =========+=========+=========+=========+=========+=========+
Ec-02_006140     -----------------------------------------------------MSKADAV
MSTRG.31196.1    MRSWSAGNGGQRRDSHVGGAGMGVFVGLFPAADSDSEDEQDRHVYHHNARAAPAAKTSKA
                                                                        #####


                         70        80        90       100       110       120
                 =========+=========+=========+=========+=========+=========+
Ec-02_006140     GGWSLFPDKE-EIPAQVGAALESGEGNHSNETSAATPGVAVRPQNSFN--SPPVGEQGSD
MSTRG.31196.1    NVWSLFPDKDGEALSQPAGASAAGGG--RSRRDGAPPGVASRMWRSFSPTKEKVAGEPSA
                 #########  ###############        #############      #######


                        130       140       150       160       170       180
                 =========+=========+=========+=========+=========+=========+
Ec-02_006140     RLSLGRGKQNEKKGVALLKSKAARS--PLRSGVHFKEGDDLEAARG-TQPPT-----TVG
MSTRG.31196.1    SSRVGRKTKQKRKVESQKEHQNMTVPSLVRAGIDEKEADDVEAARCRTRSPSASRSLSPG
                 #############                ################              #


                        190       200       210       220       230       240
                 =========+=========+=========+=========+=========+=========+
Ec-02_006140     AMPGEAANDEFELNEDRSPAEWMRVGDTTGDAFLWYTVMLEIASACCSIVYITSHDEDQL
MSTRG.31196.1    APPDTEASYGFELNEDRDPADWMRVGDTTGIAFLWFTVMLQITTACCSIIFLASDGENQL
                 ############################################################


                        250       260       270       280       290       300
                 =========+=========+=========+=========+=========+=========+
Ec-02_006140     LWKTDCWGRWTSLSDAEDVQFVEFDTHIGWQQYGKVDKLGLLSELSESTAVMGSLAGGMH
MSTRG.31196.1    SWKTGCWGMWTSLSDAENIEFVEFDTHIGWQLYGKGDKLGLLSEVSDRAAVVGGLAGGMH
                 ############################################################


                        310       320       330       340       350       360
                 =========+=========+=========+=========+=========+=========+
Ec-02_006140     GLVIIIACVFFFFSSSALWLSYFDFCCSRQ--TPTHEGKVGPKEGQKTKPAMRDHPLVLA
MSTRG.31196.1    TLVVFIACGFLFFSLSALVLTYASFFCAWQGSSSSKEGSMEAGKGARIESALPRSPLVLA
                 ##############################      ########################


                        370       380       390       400       410       420
                 =========+=========+=========+=========+=========+=========+
Ec-02_006140     RDCHRWMFVMAASYILAGLWCFGVGCIGFYVVIMGDFTDGLQDTISGWGESVSCTEPQTS
MSTRG.31196.1    KDCHRWLFAMAAIDIFAGLWCFGVGCAGFYLVVTRDLTQGLENIITGWGESVSCAKPQTS
                 ############################################################


                        430       440       450       460       470       480
                 =========+=========+=========+=========+=========+=========+
Ec-02_006140     PRYGFFANGLSILLFFGGGTIILYKTYVSDRELSEAVEMWKNDRAEMAEMEARLAMIRKK
MSTRG.31196.1    PRYGFFVNGLSVLLLLGGGIISLFKTYASHRDLKEAMEMWKNDRAEVAEMQSRLAMIRKK
                 ############################################################


                        490       500       510       520       530       540
                 =========+=========+=========+=========+=========+=========+
Ec-02_006140     EDYKAQVASAAASAAS-PVPIFKSRKSQNNAPSSFSPSPTAASSRNGYHANPSDLDDMLP
MSTRG.31196.1    EEYAAAVASAAASAAESPAIIFKSRKSVTSAMT---------------------------
                 ###############  ##############                             


                        550       560       570       580       590       600
                 =========+=========+=========+=========+=========+=========+
Ec-02_006140     LSPPDLVSPPSSAGTASTAEGWDGRHKRMATKAAFSPKDLEHDEQSVGSTADTPRDPRQS
MSTRG.31196.1    -----VPAAPAAVPAADPAE-----------------------ESSSG---------RSS
                          ###########                                        


                        610       620       630       640       650       660
                 =========+=========+=========+=========+=========+=========+
Ec-02_006140     PSLSFLESSLLQSPEPQVSNIDRDLELGSGSAAAPVDDDLPPPPGWEVRTTPMGDKFWVN
MSTRG.31196.1    RSCSPLDX----------------------------------------------------
                                                                             


                        670
                 =========+==
Ec-02_006140     IATKNVSWDRPT
MSTRG.31196.1    ------------
```

```
Parameters used
Minimum Number Of Sequences For A Conserved Position: 2
Minimum Number Of Sequences For A Flanking Position: 2
Maximum Number Of Contiguous Nonconserved Positions: 8
Minimum Length Of A Block: 10
Allowed Gap Positions: None
Use Similarity Matrices: Yes
```

```
Flank positions of the 9 selected block(s)
Flanks: [56  69]  [72  86]  [95  107]  [114  133]  [150  165]  [180  330]  [337  495]  [498  511]  [550  560]  

New number of positions in Ec-02_006140.aa.fasta.trans.aa_ali.fasta-gb:  413  (61% of the original 672 positions)
```
